# Relative and Absolute Reliability of a Motor Assessment System Using KINECT^®^ Camera

**DOI:** 10.3390/ijerph17165807

**Published:** 2020-08-11

**Authors:** Gracia Castro-Luna, Diana Jiménez-Rodríguez

**Affiliations:** Department of Nursing, Physiotherapy and Medicine, University of Almeria, 04120 Almeria, Spain; djr239@ual.es

**Keywords:** motor evaluation, reliability, Kinect camera

## Abstract

(1) Background: Virtual reality is currently useful in different clinical specialties as a diagnostic and therapeutic tool. In this study, we analyzed the relative and absolute reliability of the motor evaluation with the Kinect camera, a markerless motion system. (2) Methods: Observational study in healthy people, whose inclusion criteria were: healthy people, age 18 to 40 years old without pathologies or injuries in osteoarticular structures or ligamentous muscle and pharmacological treatment with influence on motor skills. Fifty-two subjects were evaluated. (3) Results: The results of the relative reliability were favorable in variables such as the amplitude of passage of the right leg (ICC (Intraclass Correlation Coefficient) = 0.95 ± 0.03), the step width of the left leg (ICC = 0.92 ± 0.04) or balance of the left leg (ICC = 0.90 ± 0.05). Moderate values were found for other variables. The absolute reliability, measured by the coefficient of variation, was favorable in most of the variables. (4) Conclusions: The results reflect a favorable intraclass correlation in the evaluation of the variation and asymmetry of movements of the upper limbs, the balance of both legs, the side step width and the evaluation of the sitting and standing positions. The reliability of the evaluation of the variation of movements and the asymmetry of the lower limbs must be further improved.

## 1. Introduction

In recent years, there has been a technological revolution, in which increasingly sophisticated devices and appliances have been gradually incorporated into every aspect of our daily lives. The health sector has been no stranger to this revolution, which has brought significant developments and improvements to the area. Among its many advances, therapies making use of new technologies have emerged. Using virtual reality, people can interact with a representation of the real world through a series of devices that help the user to feel fully immersed in it [1]. Aside from its recreational purpose, attempts have also been made to use virtual reality for rehabilitation. Authors such as Pearson and Mitchell [2] and Lozano-Quilis et al. [3] described a series of advantages related to virtual reality, such as the chance to interact with a three-dimensional world in which the patients can move around, the possibility to work with stimuli that do not cause harm or damage and finally the technology’s non-invasive nature. Swetteham et al. created a stable, familiar and predictable environment for children with autism that is highly adaptable to their requirements [4]. Regarding autism, the examined studies suggest moderate evidence about the effectiveness of VR (Virtual Reality)-based treatments in ASD (Autism Spectrum Disorder). VR can add many advantages to the treatment of ASD symptomatology, but it is necessary to develop consistent validation in future studies to state that VR can effectively complement traditional treatments [5]. 

Concerning the effectiveness of this type of rehabilitation in patients with movement impairments, several studies have shown favorable results regarding the use of virtual reality as a tool for rehabilitation [6,7,8]. In a study by Webster et al. [9], virtual reality was used to help subjects in wheelchairs to improve control over their mobility. After the intervention, the results showed a decrease in wheelchair-related accidents compared with a control group.

Bao et al. [10] discovered that after training with the 360Kinect^®^ (virtual reality system) (Microsoft Corp., Redmond, WA, USA) acute stroke patients exhibited a significant improvement in upper limb function. Changes in primary sensorimotor cortex activation were also found, indicating an enabling of brain neuroplasticity after the accident. Furthermore, specialists in neurorehabilitation have encountered increased patient interest when performing exercises in virtual reality; users found it more entertaining than performing traditional exercises, and did not encounter any side effects. The 360 Kinect^®^ is a simple, low-cost, portable measurement procedure, which does not require marks to be placed around the body to take measurements, instead interpreting 3D body posture in real-time. After Bao’s study, researchers began to study the precision and accuracy of virtual reality devices in rehabilitation of patients with movement impairments and use them as a tool to evaluate patients’ progress [11,12,13,14].

Authors such as Yeung et al. [15] and Tanaka et al. [16] evaluated the 360 Kinect^®^ as a clinical assessment tool for the center of mass. They evaluated four maneuvers and compared them with the Vicon system (marked motion system with multiple cameras around the subject). They found that the 360 Kinect^®^ showed potential as an assessment tool for the center of mass. The 360 Kinect^®^ showed excellent reliability and validity. A motor assessment software was designed using clinical maneuvers for the detection and monitoring of motor impairments. This software aimed to improve this process, enabling information to be gathered more quickly and facilitating patient data collection [17]. The main objective of this study was to analyze the relative and absolute reliability of a motor assessment based on the exploration of maneuvers defined as non-complicated movements or simple biomechanical patterns, measured through 360 Kinect^®^ kinematic analysis to be used in patients with movement impairments who need an objective quantifiable follow-up of their progression. We preferred to conduct the evaluation in healthy people so as to quantify and detect any variation in the measurement. A secondary objective was to evaluate the influence of physical activity levels on motor evaluation results to find a correlation between physical activity and gross motor development [18,19,20].

## 2. Materials and Methods

### 2.1. Study Design

Cross-sectional study.

### 2.2. Settings

Fifty-two subjects were recruited from January to March 2019. Only 29 subjects completed the evaluation period.

### 2.3. Participants

The inclusion criteria were: healthy men and women aged between 18–40 without illness or injuries to osteoarticular, muscle or ligament structures in the previous six months and not undergoing any drug treatments that would impact motor skills.

### 2.4. Study Size

The sample was composed of 18 men and 11 women, with an average age of 24 (Table 1). All subjects gave their informed consent for inclusion before they participated in the study. The study was conducted in accordance with the Declaration of Helsinki, and the Ethics Committee approved the protocol 46/2018 (Ethical Committee of the Department of Nursing, Physiotherapy and Medicine, University of Almeria).

### 2.5. Data Measurements

#### 2.5.1. Anthropometric Measurements:

An electronic scale was used to measure body mass (Jata, 555, A-4250) to the nearest 100 g. For stature, a stadiometer with a maximum length of 210 cm placed on the wall (Sohenle professional, 5002.01, Germany) was used. The body mass index (BMI) was calculated by dividing body mass (in kilograms) by stature (in meters) squared.

#### 2.5.2. Measurement of Physical Activity Levels

The subjects were classified as “active” (n = 16) or “sedentary” (n = 16). To do so, the guidelines regarding minimum weekly physical activity levels set out by the World Health Organization (WHO) of at least 150 min a week of moderate-to-vigorous physical activity carried out in at least 10 min blocks.

#### 2.5.3. Description of the 360 Kinect^®^ System

A 360 Kinect^®^ (Microsoft Corp., Redmond, WA, USA) motion-capture camera system was used to measure motor functions.

The hardware consists of an infrared depth sensor and a red, green and blue (RGB) image sensor camera to capture motion in 3D video. A virtual skeleton model, composed of 20 points, can be directly obtained through the 360 Kinect^®^ v1.8 Software Development Kit (SDK). The sampling frequency of the 360 Kinect^®^ is 30 Hz, and the camera resolution is 640 × 480 pixels.

#### 2.5.4. Motion Capture

Several studies [20,21] analyzed the reliability, accuracy, and validity of the 360 Kinect^®^ system as a tool to capture movement, comparing it to the “gold standard” Vicon System. An exact location of the virtual skeleton in the three coordinates of space is necessary to be able to evaluate its movement accurately. The results showed that the system is suitable for the capture of coordinates with an average error measurement and standard deviation of 0.0065 m (±0.0048), 0.0109 m (±0.0059) and 0.0057 m (±0.0042) in the x, y and z axes, respectively. The system produces a precision in the range 1.0 to 3.0 m from the camera and a 54.0° horizontal and 39.1° vertical field of vision [21,22]. The subjects had to wear sporty, tight-fitting clothing, avoiding reflective colors or any other garments that could decrease infrared accuracy [21].

#### 2.5.5. Motion Assessment

Motion assessment was based on the exploration of maneuvers described below. Before carrying out the maneuvers, the subject placed him/herself in front of the 360 Kinect^®^ for calibration. This process consisted of framing the subject correctly in the center of the machine’s field of vision. The subject was required to do some arm abductions to check that the body was completely captured by the camera while leaving a small gap on each side. The entire process was performed under the guidance and supervision of the researchers. Up to two practice rounds were completed before measurements being taken to minimize “the learning effect”.

The participants were instructed to carry out a standardized “greeting” similar to “a bird flapping”. This movement was performed before each maneuver to mark the beginning of each maneuver, facilitating data extraction by the researchers.

Following this pattern, the subject completed three rounds of six maneuvers. Each maneuver was repeated five times except static positions, such as the T-position test or resting position test. This procedure was designed to minimize errors about the performance of each maneuver and improve the accuracy of its realization; it is particularly important in cases of patients with impaired movement. All of the maneuvers included in the software can be found in neurological scales in primary care, neurological scans or motor state assessment tests [23,24].

✓ The resting test (RT) consisted of maintaining a standing position with the arms resting at the side of the body for approximately 10 s.
✓ The T-position test (TT) was conducted in a standing position, with the arms outstretched horizontally at a 90° angle from the body. This position was held for about 10 s.
✓ The finger-to-nose test (FNT) with opened eyes, participants stood with their arms outstretched horizontally at a 90° angle from the body. They then had to touch the tip of their nose with alternating index fingers, returning to the original position between each movement. Before starting any movement with the opposite hand, they had to return to the original position fully. This maneuver was repeated five times with each arm.
✓ The sideways step test (SST) started from the initial resting position with the arms at the sides of the body. Participants then took a step sideways with their left foot, reaching as far as they could but without the movement causing them to lose balance. They then returned to the resting position by stepping back in the opposite direction with the same foot. After this, they repeated the process with the right foot. This maneuver was repeated five times with each foot.
✓ The monopodal static balance test with 45° leg abduction (T45) started from the initial resting position with the arms at the sides of the body. Participants then had to raise their left foot to the side, trying to reach an angle between the legs of approximately 45°. This position was held for 10 s, subsequently lowering the foot gently back to the initial position. Once this movement was complete, the maneuver was repeated with the right foot.
✓ For the chair at 90° to standing test (CST), a fixed, hard armless chair was used, adjusted in stature. Before carrying out the maneuver, the chair was adjusted so that the hips and knees were at the same level, parallel to the ground, thus forming a 90° angle. This maneuver started from the initial standing position. The subjects then had to sit down in the most natural way possible without using their arms for support. After that, they had to return to the original standing position. The full maneuver was repeated five times.

After the subject had completed one series, a resting period of about three minutes was given to avoid a possible build-up of fatigue that could alter the measurements. After this time, the subject went on to start a new series. Three series were completed.

#### 2.5.6. Data Collection

Video capturing was carried out using the 360 Kinect^®^ SDK with a PC set up for these types of calculations, with a 8.1 Pro Windows system (©2013 Microsoft Corporation), an Intel(R) Core (TM) system processor (i5-440 CPU @ 3.10 GHz), 8.00 GB RAM and a 64 bit operating system. The figure was segmented, extracting crucial information in the form of a graph (“skeleton”), consisting of nodes (joints) and connections. The file was subsequently converted into a compatible format so that it could be interpreted by the MATLAB mathematics program^®^ (R2013a). With these data, the time series (X, Y and Z coordinates) were recorded at each point of the skeleton during the maneuver. The primary measures (distances between points at any given moment, angles and angular velocities) were identified and used to calculate the necessary measurement type for each body segment in each test.

Finally, the data were integrated to calculate the different variables for each motor skills test.

### 2.6. Variables

Variables were continuous except sex and physical activity levels. The continuous variables were as follows. Stature and variables of motor assessment; accuracy of movement: left/right arm (AccMov_LA, AccMov_RA); speed of movement: left/right arm (Veloc_LA, Veloc_RA); variation of movement: left/right arm (VarMov_LA, VarMov_RA); involuntary movement: left/right arm (InvMov_LA, InvMov_RA); variation of movement: left/right leg (VarMov_LL, VarMov_RL); static monopodal balance: left/right leg (MonSt_Bal_LL, MonSt_Bal_RL); amplitude of step: left/right leg (AmpStep_LL, AmpStep_RL); variation of axial movement (Var_Mov_axial); axial stability (Stab_axial); upper limb asymmetry (Asymmetry_UL); lower limb asymmetry (Asymmetry_LL); resting position (RT); T-position test (TT); finger-to-nose test (FNT); sideways step test (SST); chair at 90° to standing test (CST); monopodal static balance test with 45° leg abduction (T45). The characteristics of these variables are described in Table 2.

### 2.7. Statistical Analysis

All of the statistics were carried out using IBM SPSS version 25 (IBM Corp., Armonk, NY, USA); the significance level was α = 0.05.

One-factor repeated measures ANOVA analysis was used to calculate relative reliability (internal consistency). It was determined through the intraclass correlation coefficient (ICC) and its 95% confidence interval between the three measurements on the same day [25]. The ICC was categorized as follows: values of 0.50 to 0.69 were considered “moderate”, 0.70–0.89 were considered “high” and 0.90 and above were considered “excellent” [26].

Absolute reliability was determined by calculating the standard error of measurement (SEM), using the square root of the intra-subject root mean square. The coefficient of variation (CV) was calculated through the division of the SEM by the sum of the attempt average multiplied by 100 [26]. In the literature, values of 10% or less are suggested, somewhat arbitrarily, as acceptable for the CV [26].

A one-factor repeated-measures ANOVA was conducted to quantify the mean difference between the three attempts. For contrast, the variables were checked to determine the sphericity assumption through Mauchly’s sphericity test (1940). If sphericity was determined, the F univariate was used. The F degrees of freedom were adjusted by multiplying by the estimated Epsilon value in other cases. The sample effect size (ES) was calculated using Hopkins’ spreadsheet. Determining the existence of differences between variables was carried out to qualitative measurements: a difference was considered to be substantial when the valuation was “likely” or higher than 75%, and also when the effect size (<0.41 (small); 0.41–0.70 (moderate); ≥0.70 (large)) was moderate or large.

Finally, for the second hypothesis analyses, the sample was organized into two groups according to levels of physical activity. Depending on the parameters of normality, homoscedasticity, randomness and independence, either Student’s statistical t-test or the Wilcoxon–Mann–Whitney test were performed.

### 2.8. Bias

The sample of participants was small. They were all young participants so the differences in terms of their physical activity were not significant.

## 3. Results

Table 2 includes the demographic characteristics of the sample. Table 2 shows the results of the average difference between attempts of the upper limbs. High values of reliability were observed in other variables, such as posture, sitting down and standing up, right leg balance, asymmetry of upper limbs, asymmetry of lower limbs, left arm movement accuracy and upper limbs variation of movement. Moderate values of relative reliability in left arm speed, right arm speed, left arm involuntary movement and right leg variation of the movement were also found (Table 3).

Excellent reliability values were found in variables including width of right leg step (ICC = 0.95), width of left leg step (ICC = 0.92) and left leg balance (ICC = 0.90). Regarding absolute reliability, coefficient of variation values (below 10%) were found in posture when sitting down and standing up, axial balance, left leg balance, left arm movement accuracy, upper limbs speed and left arm involuntary movement. Data close to acceptable (around 10%) were found in the width of the right leg step variable (Table 4).

The averages of the evaluations were compared. No significant differences were found between the attempts of any of the variables (Table 5).

The average of the three assessments was established as the representative value for the attempts. The subjects were divided into “active” and “sedentary”. None of the variables analyzed showed significant differences between the two groups studied (Table 6 and Table 7). We already assumed this homogeneity since the maneuvers explored were simple movements whose only purpose was to detect deficiencies but not the physical form of the patient, which was not relevant in this evaluation. Even so, it was necessary to evaluate if there could be differences according to their physical conditions to assess the stability of the exploration.

If we take into account the orientation of the evaluation towards patients with movement deficit, this homogeneity increased its validation and the results as an evaluation of elementary movements did not depend on previous physical activity.

## 4. Discussion

Investigations into motor function and musculoskeletal health will require easily obtainable, valid and reliable measures of gross motor function and kinematics. Marker-based motion capture systems provide reasonably valid and reliable measures, but recordings are restricted to expensive lab environments. Markerless motion capture systems can provide measures of gross motor function and kinematics outside of lab environments and with minimal interference to the subjects being investigated. It is, however, unknown if these measures are sufficiently valid and reliable in healthy people to warrant further use. This study aimed to evaluate the markerless motion capture based on the 360 Kinect^®^ system in relation to other established systems. We evaluated twenty variables based on six maneuvers. Our results demonstrated the relative and absolute reliability of a gross motor assessment system using the 360 Kinect^®^ system with healthy people. High values of reliability were observed in variables such as posture when sitting down and standing up, right leg balance, asymmetry of upper limbs, asymmetry of lower limbs, left arm movement accuracy, upper limbs variation of movement, width of right leg step (ICC = 0.95), width of left leg step (ICC = 0.92), and left leg balance (ICC = 0.90). Moderate values of relative reliability were found in left arm speed, right arm speed, left arm involuntary movement and right leg variation of the movement. As mentioned above, moderate to high values of both absolute and relative reliability were found for the majority of the analyzed variables. Furthermore, no differences in motor abilities were observed in terms of physical activity levels in the analyzed sample.

The 360 Kinect^®^ camera showed moderate to excellent reliability in terms of internal consistency (values over 0.70) in nine out of the 20 variables studied. However, six variables with an ICC below 0.50 were also found.

These results contrast with those found by other authors in different studies. Huber et al. [11] found ICC values of between 0.76 and 0.98 in different shoulder joint movements with the Kinect^®^ camera. Similarly, Bonnechere et al. [12] studied single shoulder, elbow, knee and hip movements (such as abduction and flexion), obtaining ICC values between 0.70 and 0.84. Another study analyzed the reliability of the Kinect^®^ camera when evaluating foot posture using the foot posture index (FPI) test methodology, adapting it to the Kinect, and obtained intrasubject reliability of between 0.62 and 0.78 [13].

Nevertheless, there were also studies with results similar to those found. Clark et al. [14] measured medial-lateral and anteroposterior balance in static and dynamic balance tests, finding ICC values of between 0.18 and 0.91. This suggests that if we move away from simple measurements (such as flexions, stretches, abductions or adductions) to other more complex ones involving multiple nodes and multiple planes and even at different times in an integrated manner (like those analyzed in this study), the ICCs have a greater spread, ranging from very low to very high reliability. This could be due to the data integration algorithm used to obtain data in the Matlab program.

Another possible way to justify the findings could be the difficulties encountered by 360 Kinect^®^ in carrying out measurements for certain joints of the body, especially in the more distal joints, such as hands or feet. The 360 Kinect^®^ image resolution (640 × 480 pixels) is slightly lower than that of other motion detection systems, which can reach 2352 × 1728 pixels [15]. Low accuracy and sensitivity in the depth sensor could also explain low levels of reliability [22]. Furthermore, in this study, the maneuvers were carried out with the palms of the hands facing the floor in a horizontal position. It is probable that if the maneuvers were performed with open palms facing the Kinect^®^ lens, there would be a larger surface area to recognize these points, and thus they would be better detected.

In a recent study, Tanaka et al. [16] calculated the center of gravity (COG) of 18 healthy adult participants. The coordinates of the joint centers during the sit to stand (STS) motion were collected using the 360 Kinect^®^ as an MLS (Markerless System) and the Vicon system (as a marker-based motion capture system (MBS)). The centers of mass of each segment, which were calculated based on the segmental mass and length, were synthesized to calculate the COG. The displacement, velocity and acceleration of the COG during the STS motion were calculated from the data obtained using each system and compared between systems. The results of both systems were similar.

Regarding the differences between the “active” and “sedentary” groups, no significant differences were found in any variables.

These results do not agree with those found by multiple authors in the scientific literature among young and old age groups. These studies found positive correlations between the level of physical activity and gross motor skills [18,19,20].

The difference between physical activity and motor assessment results could be explained by the homogeneity of the subjects’ characteristics. Another possible cause may be the simplicity of the maneuvers themselves, designed to analyze people’s motor function status.

In the scientific literature, significant differences have been found in a population with some type of motor disorder (e.g., Stroke, Cerebral Paralysis, Parkinson’s, Multiple Sclerosis), and some researchers have shown how motor skills improve after an intervention period using virtual reality [3,7,11,12]. These authors used different scales and balance tests such Tinetti or Berg, the Community Balance and Mobility Scale, the Sensory Organization Test or the Bruner Assessment Balance; for upper limb functionality the Fugl–Meyer Assessment scale; or the Gross Motor Function Measure Dimension for general motor status [11,12]. Many of these scales share maneuvers and variables similar to the ones included in our study, and differences depending on physical activity levels in populations with disorders.

A large and significant number of studies is needed to demonstrate these differences. The improvement of psychometric data would probably be influenced by the limitations of the measuring instrument.

Despite its benefits, the 360 Microsoft Kinect^®^ system shows an inability to assess multiple internal/external rotations and difficulty measuring in the more distal joints. These flaws are restrictive, limiting the angular data of the distal joints in flexion/extension and abduction/adduction [17]. This restriction could have an influence on data collection and therefore data reliability. Calibration equations could be generated to correct these constraints, and future versions of the SDK could be designed to help obtain significantly better results [17].

Some of the weaknesses previously encountered regarding the 360 Kinect^®^ system were overcome in this study. Huber et al. [11] found that the execution of certain maneuvers, such as shoulder flexion at 90°, affected the reliability due to the occlusion of certain joints. In our case, this maneuver was replaced by a shoulder abduction at 90°, enabling the camera to acquire the data more efficiently. The same procedure was followed with the balance in both legs, in this case using abduction.

Other markerless motion system are the “Captury” motion system, “The Opto Gait” [27] and the Dynamic Athletic Research Institute (DARI) system. DARI (Motion Platform, version 3.2-Denali, from Scientific Analytics Inc., Kansas City, KS, USA) is an advanced tool that consists of a powerful cloud processing software engine that takes thousands of data points comprising each motion and processes them in less than a second. Most of the published studies that use the DARI system and other markerless systems were in the field of sports medicine (Parkinson’s disease), for professional athletes, rehabilitation and injury prevention. Martinez et al. evaluated the relationships between DARI parameters and the UPDRS (Unified Parkinson’s Disease Rating Scale) [28]. Cabarkapa et al. [29], using the DARI motion system, performed a standardized 19-movement test designed to assess overall body functional motor capabilities. For each movement during the test, both devices were recording and collecting data simultaneously. System algorithms used 192 test variables to calculate five scores (arbitrary units): power, functional strength, dysfunction, composite (power + functional strength − dysfunction) and vulnerability.

The problem with different markerless motion systems is that the biomechanical patterns they use are different, and thus the results cannot be compared or discussed. For example, Harsted et al. [30] examined the following variables with the Captury motion system: jump length, jump height, hip flexion, knee flexion, ankle dorsiflexion, knee varus, knee to hip separation distance ratio (KHR), ankle to hip separation distance ratio (AHR), frontal plane projection angle, frontal plane knee angle (FPKA) and frontal plane knee deviation (FPKD). Even Tanaka et al. [16], using the 360 Kinect^®^ system, calculated the displacement, velocity and acceleration of the COG (center of gravity) during STS (sit to stand) motion; however, their results are not comparable with the variables in our study.

The global and integrated visions of motor status should be highlighted as strengths of the study. The majority of authors measured precise movements (pelvic movements, range of shoulder or ankle movement, trunk movements) [31,32]. By contrast, in our case, these were integrated into larger constructs such as balance, providing a more global view of the patient’s state, despite the need to improve data reliability. Our study also represents significant progress in the study of virtual reality for health care, specifically in the field of clinical evaluation.

However, several study limitations were also detected. One of the main limitations is related to the calculation of relative and absolute reliability. The study was carried out with a healthy population, as it was impossible to access a homogeneous group of people with a motor disorder; the results would therefore not be comparable with such a population. Another limitation of the study lies in the difficulty to control the speed of maneuver execution. The subjects had freedom with these movements, and the only instruction from researchers was to move at an “average speed”. Segmentation of the videos for conversion to the Matlab format was done manually by the researchers. A start for each maneuver was established through the “greeting”, and the approximate 10 s guideline only determined the end of some maneuvers, which were quantified by execution time (resting maneuver, T-test and balance test with the foot at 45°). Without a good systematized video cutting system, this had to be done manually frame by frame, with the consequent errors it may entail.

There is a vast field of potential lines of future research that would complement and build upon this study.

## 5. Conclusions

The data provided by the 360 Kinect^®^ system regarding motor function assessment are promising for evaluating movement. The results reflect an excellent intraclass correlation in the evaluation of the variation and asymmetry of movements of the upper limbs, the balance of both legs, the side step width and the evaluation of the sitting and standing positions. Reliability of the variation of movements and the asymmetry of the lower limbs must be improved.

## 6. Patents

Patents resulting from the work reported in this manuscript: Neurobia software for movement evaluation: CA-388-15. Registration number: 201599902505082.

## Figures and Tables

**Table 1:** Motor function tests: movement parameters calculation.

Variables	Description of Measurement Type for Calculation	Motor Function Tests
AccMov_LA AccMov_RA	Accuracy touching the nose with left/right arm and accuracy of left/right arm returning to the T position. Maximum range of movement of the hand in T position, the variability of left/right arm movement when touching the nose and Left/right arm tilt angle returning to T (arm drop).	(FNT)
Veloc_LA Veloc_RA	Average speed of left/right arm movement to the nose and average speed of left/right arm movement to the T position.	(FNT)
VarMov_LA VarMov_RA	Left/right arm tilt angle returning to T (arm drop) and variability of left/right arm movement when touching the nose.	(FNT)
InvMov_LA, InvMov_RA	The maximum range of movement of the resting hand. The average speed of movement of the resting hand. The maximum range of movement of the hand in T position. The average speed of movement of the hand in T position.	(RT), (TT)
VarMov_LL VarMov_RL	The average height reached by the foot. Percentage of time that foot remained raised—dispersion of width of step to the right.	(SST)
MonSt_Bal_LL MonSt_Bal_RL	Time foot was kept in the air at 45 ° during the test. Flexion of the leg in the air during the position.	(T45)
AmpStep_LL AmpStep_RL	Width of left/right step (angle between the legs).	(SST)
VarMov_Axial	Dispersion over five repetitions of the hip position to sit down and stand up.	(CST)
Stab_Axial	The maximum range of movement of the head in resting position. The average speed of movement of the head in resting position. The maximum range of movement of the head in T position. The average speed of movement of the head in T position.	(RT), (TT)
Sitting down and standing up posture	Average speed to sit down and stand up and displacement angle of the trunk in the frontal plane.	(CST)

Accuracy of movement: left/right arm (AccMov_LA, AccMov_RA), Speed of movement: left/right arm (Veloc_LA, Veloc_RA), Variation of movement: left/right arm (VarMov_LA, VarMov_RA), Involuntary movement: left/right arm (InvMov_LA, InvMov_RA), Variation of movement: left/right leg (VarMov_LL, VarMov_RL), Static monopodal balance: left/right leg (MonSt_Bal_LL, MonSt_Bal_RL), Amplitude of step: left/right leg (AmpStep_LL, AmpStep_RL), Variation of axial movement (Var_Mov_axial), Axial stability (Stab_axial), Upper limb asymmetry (Asymmetry_UL), Lower limb asymmetry (Asymmetry_LL). Resting position (RT), T-position test (TT), finger to nose test (FNT), sideways step test (SST), chair at 9\0º to standing test (CST), monopodal static balance test with 45° leg abduction (T45).

**Table 2:** Subject characteristics.

Characteristics	(n = 29)	%	Mean (±SD)
Age (years)			23.9 (±5.1)
Men	18	62.06	
Women	11	37.9	
Stature (cm)			170.74 (±9.21)
Weight (kg)			72.9 (±12.7)
Body Mass Index (BMI)			24.9 (±3.4)
>25	18	62.06	
25–30	8	27.5	
>30	3	10.44	

**Table 3:** The average difference between attempts—upper limbs.

Endpoints	Attempts							
	Assessment 1 Mean ± SD	Assessment 2 Mean ± SD	Assessment 3 Mean ± SD	df	F	Sig.	ES	IC (95%) Limits
Accuracy of movement: left arm	5.23 ± 2.77	5.14 ± 2.50	5.30 ± 2.14	1.48 ^^^	0.56	0.899	(0.00) ± 0.36	(−0.37; 0.36)
Accuracy of movement: right arm	5.07 ± 1.42	4.74 ± 1.27	4.89 ± 1.31	2	0.57	0.567	(−0.22) ± 0. 47	(−0.69; 0.25)
Left arm speed	7.31 ± 0.97	7.15 ± 0.90	7.25 ± 0.62	2	0.32	0.728	(−0.14) ± 0.44	(−0.58; 0.30)
Right arm speed	7.23 ± 0.96	7.09 ± 0.80	7.10 ± 0.66	1.37 ^	0.45	0.568	(−0.13) ± 0.35	(−0.48; 0.22)
Variation of movement: left arm	1.07 ± 0.52	1.14 ± 0.50	1.17 ± 0.53	2	0.78	0.466	(0.15) ± 0.27	(−0.13; 0.42)
Variation of movement: right arm	0.62 ± 0.32	0.64 ± 0.28	0.59 ± 0.23	2	0.88	0.420	(0.11) ± 0.21	(−0.10; 0.33)
Involuntary movement: left arm	7.26 ± 1.74	7.09 ± 1.82	7.37 ± 2.07	2	0.26	0.775	(−0.12) ± 0.47	(−0.59; 0.35)
Involuntary movement: right arm	8.47 ± 2.89	8.10 ± 3.57	8.05 ± 1.91	2	0.22	0.805	(−0.24) ± 0.54	(−0.77; 0.30)

Sphericity is not met and Greenhouse–Geisser is used (^). Univariate F statistic. Degrees of freedom (df). *p*-value < 0.05, CI (95%), ES reference values; Cohen (<0.41 (low); ≥0.41–0.7 (moderate), >0.7 (high)).

**Table 4:** The average difference between attempts—lower limbs.

Endpoints	Attempts							
	Assessment 1 Mean ± SD	Assessment 2 Mean ± SD	Assessment 3 Mean ± SD	df	F	Sig.	ES	IC (95%) Limits
Variation of movement: left leg	8.18 ± 1.96	7.51 ± 1.41	7.62 ± 1.49	2	1.98	0.148	(−0.33) ± 0.40	(−0.73; 0.07)
Variation of movement: right leg	8.86 ± 2.42	8.36 ± 1.87	8.13 ± 2.14	2	1.43	0.248	(−0.17) ± 0.39	(−0.56; 0.23)
Balance: left leg	47.59 ± 8.10	47.45 ± 8.76	47.10 ± 8.76	2	0.26	0.770	(−0.06) ± 0.21	(−0.27; 0.15)
Balance: right leg	37.99 ± 6.92	37.69 ± 7.04	36.50 ± 5.55	1.42 ^	1.11	0.320	(−0.05) ± 0.38	(−0.43; 0.32)
Side step width: left leg	27.65 ± 6.95	26.80 ± 6.30	28.33 ± 7.76	2	1.77	0.179	(−0.12) ± 0.24	(−0.37; 0.12)
Side step width: right leg	27.47 ± 7.84	26.87 ± 6.80	28.04 ± 6.69	2	1.46	0.242	(−0.07) ± 0.18	(−0.25; 0.11)
Variation of axial movement	2.30 ± 1.00	2.07 ± 1.13	2.12 ± 1.22	2	0.50	0.606	(0.30) ± 0.40	(−0.71; 0.10)
Axial balance	2.99 ± 0.46	3.01 ± 0.34	2.95 ± 0.32	2	0.40	0.671	(0.09) ± 0.37	(−0.28; 0.46)
Axial stability	3.20 ± 0.70	3.36 ± 0.80	3.18 ± 0.80	2	0.57	0.567	(0.21) ± 0.46	(−0.25; 0.66)
Posture: sitting down and standing up	3.06 ± 0.39	3.63 ± 0.39	3.63 ± 0.39	1.48 ^	0.43	0.916	(−0.07) ± 0.25	(−0.19; 0.32)
Upper limb asymmetry	8.39 ± 2.63	7.83 ± 2.09	7.44 ± 2.66	2	2.29	0.111	(−0.19) ± 0.37	(−0.56; 0.19)
Lower limb asymmetry	19.90 ± 5.24	20.56 ± 4.64	20.78 ± 6.13	2	0.39	0.677	(−0.13) ± 0.37	(−0.24; 0.50)

Sphericity is not met, and Greenhouse–Geisser is used (^). Univariate F, statistic. Degrees of freedom (df). *p*-value < 0.05, CI (95%), ES reference values; Cohen (< 0.41 (low); ≥ 0.41–0.7 (moderate), > 0.7 (high)).

**Table 5:** Results of reliability.

	F	Sig.	ICC	CI (95%)	SEM	CV (%)
Accuracy of movement: left arm	0.07	0.934	0.74	(0.523; 0.873)	0.47	8.99
Accuracy of movement: right arm	0.82	0.448	0.40	(−0.13; 0.70).	1.03	20.90
Left arm speed	0.31	0.738	0.51	(0.09; 0.76)	0.40	5.60
Right arm speed	0.81	0.449	0.67	(0.38; 0.83)	0.57	8.02
Variation of movement: left arm	0.76	0.472	0.88	(0.78; 0.94)	0.25	22.13
Variation of movement: right arm	0.84	0.439	0.89	(0.79; 0.94)	0.14	22.46
Involuntary movement: left arm	0.05	0.951	0.66	(0.37; 0.83)	0.33	4.62
Involuntary movement: right arm	0.40	0.675	0.38	(−0.16; 0.69)	1.53	19.84
Variation of movement: left leg	0.59	0.556	0.48	(0.03; 0.74)	1.13	14.56
Variation of movement: right leg	0.90	0.411	0.68	(0.41; 0.84)	1.58	18.67
Balance: left leg	0.26	0.770	0.90	(0.80; 0.94)	3.12	6.48
Balance: right leg	1.15	0.335	0.80	(0.64; 0.90)	6.61	17.60
Side step width: left leg	1.61	0.210	0.92	(0.85; 0.96)	3.95	14.32
Side step width: right leg	1.48	0.237	0.95	(0.91; 0.98)	3.09	11.27
Variation of axial movement	0.51	0.606	0.47	(0.01; 0.74)	1.01	44.66
Axial balance	0.40	0.671	0.60	(0.25; 0.80)	0.29	9.71
Axial stability	0.93	0.399	0.39	(−0.13; 0.70)	0.68	20.85
Posture: sitting down and standing up	0.43	0.957	0.84	(0.70; 0.92)	0.07	1.93
Upper limb asymmetry	2.04	0.140	0.79	(0.60; 0.89)	2.37	30.05
Lower limb asymmetry	0.39	0.667	0.35	(−0.28; 0.67)	3.81	18.03

ICC: Intraclass correlation coefficient, CI: Confidence interval, SEM: Standard error of measurement, CV: Coefficient of variation.

**Table 6:** Upper limbs. Differences between sedentary and active participants.

Evaluation Variables	PA Groups (min/Week)			
	Sedentary Mean ± SD	Active Mean ± SD	*p* * Value	CI (95%) Limits
Accuracy of movement: left arm	4.52 ± 1.77	5.75 ± 2.09	0.086	(0.27; 1.48)
Accuracy of movement: right arm	4.94± 1.00	5.10 ± 0.94	0.609	(−0.40; 1.19)
Left arm speed	5.65 ± 0.82	5.64 ± 0.64	0.945	(−0.64; 0.75)
Right arm speed	7.08 ± 0.75	7.18 ± 0.58	0.706	(−0.40; 0.45)
Variation of movement: left arm	1.19 ± 0.40	1.08 ± 0.51	0.26	(−1.14; 0.31)
Variation of movement: right arm	0.63 ± 0.26	0.60 ± 0.26	0.805	(−0.98; 0.44)
Involuntary movement: left arm	7.18 ± 1.42	7.29 ± 1.49	0.84	(−0.82; 0.79)
Involuntary movement: right arm	8.37 ± 2.09	8.08 ± 1.78	0.699	(−0.71; 0.97)

* Wilcoxon-Mann-Whitney test.

**Table 7:** Lower limbs. Differences between sedentary and active participants.

Evaluation Variables	PA Groups (min/Week)			
	Sedentary Mean ± SD	Active Mean ± SD	*p* * Value	CI (95%) Limits
Variation of movement: left leg	7.63 ± 1.37	7.88 ± 1.11	0.602	(−0.49; 1.11)
Variation of movement: right leg	8.09 ± 1.92	8.72 ± 1.49	0.246	(−0.45; 1.32)
Balance: left leg	46.20 ± 10.87	49.65 ± 3.28	0.39	(−0.33; 0.87)
Balance: right leg	37.20 ± 5.65	37.81 ± 6.04	0.926	(−0.96; 1.02)
Sidestep width: left leg	24.82 ± 4.54	29.67 ± 7.20	0.051	(0.04; 2.07)
Sidestep width: right leg	25.18 ± 5.27	29.18 ± 7.48	0.127	(−0.21; 1.63)
Variation of axial movement	2.50 ± 0.60	2.07 ± 0.87	0.07	(−1.74; 0.05)
Axial balance	2.94 ± 0.25	3.01 ± 0.32	0.745	(−0.57; 0.88)
Axial stability	3.31 ± 0.55	3.20 ± 0.51	0.626	(−1.01; 0.80)
Transference	3.69 ± 0.38	3.64 ± 0.30	0.739	(−1.11; 0.63)
Upper limb asymmetry	8.17 ± 1.77	7.67 ± 2.31	0.353	(−1.26; 0.61)
Lower limb asymmetry	20.83 ± 2.51	21.38 ± 3.46	0.648	(−0.50; 1.35)

* Wilcoxon–Mann–Whitney test.
